# Towards a Molecular Classification of Colorectal Cancer

**DOI:** 10.3389/fonc.2015.00046

**Published:** 2015-02-25

**Authors:** Alessandro Lugli

**Affiliations:** ^1^Clinical Pathology Division, Institute of Pathology, University of Bern, Bern, Switzerland

**Keywords:** colorectal cancer, molecular classification, Cdx2, Wnt signaling pathway, MGMT, telomere length, Ras signaling pathway, microRNA

In colorectal cancer (CRC), an internationally standardized molecular classification has not been implemented yet. Nevertheless, there are different pathogenetic aspects that could form the basis for a future molecular classification in CRC (1).

In the classic model, CRC is divided into two major pathways. The first is the chromosomal instability pathway including 85% of all CRCs. This pathway is based on the adenoma–carcinoma sequence, which is defined by consecutive mutations in the APC, KRAS, and TP53 gene and deletion of the chromosome 18q (2). An example of an autosomal dominantly inherited disease, which belongs to the chromosomal instability pathway group, is the familial adenomatous polyposis syndrome (FAP). The second, namely the microsatellite instability pathway (15% of all CRCs) is defined by a mismatch-repair deficiency, which leads to a genomic instability. The microsatellite instability status can be sporadic (12%) or hereditary (3%, Lynch syndrome-associated CRC, HNPCC). Sporadic cases can be differentiated from hereditary cases by using the Amsterdam/Revised Bethesda criteria.

In 2007, Jeremy Jass proposed a molecular classification based on clinical, morphological, and molecular parameters, which included five subgroups (3).

Group 1 (12% of all CRCs): chromosomally stable, MLH1 methylated, MSI-H, BRAF mutated, CpG island methylator phenotype (CIMP) high status; group 2 (8% of all CRCs): chromosomally stable, partially MLH1 methylated, microsatellite stable (MSS, MSI-L), BRAF mutated, CIMP high; group 3 (20% of all CRCs): chromosomally instable, MGMT methylated, MSS/MSI-L, KRAS mutated, CIMP low; group 4 (57% of all CRCs): chromosomally instable, MSS/MSI-L, CIMP negative; group 5 (3% of all CRCs): chromosomally stable, MSI-H, CIMP negative, BRAF wild type.

Following up on this last classification, in 2010, Barbara Leggett and Vicki Whitehall published a pathogenetic overview of sporadic CRC including three pathways (4).

The serrated pathway: MSI-H, CIMP high, BRAF mutated (corresponds to the Jass group 1); the alternative pathway: MSS, CIMP low, KRAS mutated (corresponds to the Jass group 3; the traditional pathway: MSS, CIMP negative, BRAT wild type, KRAS wild type (corresponds to the Jass group 4).

In 2012, the Cancer Genome Atlas Network published a promising approach for a future molecular classification based on different pathways (5).

Group 1 (wnt and TGF-beta pathway): proliferation, stem cell, and progenitor phenotype; group 2 (PIK3CA and RTK–RAS pathway): proliferation, cell survival, translation; group 3 (p53 pathway): proliferation, cell survival. This approach may elucidatepromising target molecules for wnt pathway inhibitors or proteins of the PIK3CA and RTK–RAS pathway such as IGF2, IGFR, ERBB2, ERBB3, MEK, AKT, and MTOR.

Recently, a new proposal for a molecular classification of CRC was published by a research group from Oxford based on 906 stage II and III CRCs (6).

Group 1: MSI-H and/or BRAF mutated; group 2: chromosomally instable and/or TP53 mutated with KRAS and PIK3Ca wild type status; group 3: chromosomally instable, KRAS and/or PIK3CA mutated; TP53 wild type status; group 4: chromosomally stable, KRAS and/or PIK3CA mutated; TP53 wild type status; group 5: NRAS mutated; group 6: no mutations; group 7: other.

All these proposals for a CRC molecular classification have in common that some molecular features such as KRAS, BRAF, microsatellite status, and CIMP are often included. Nevertheless, it has to be kept in mind that molecular markers can be prognostic (i.e., BRAF), predictive (i.e., RAS), or both (i.e., microsatellite status). Therefore, the main aim of the research topic “Toward a molecular classification of colorectal cancer” was to include articles that focus on the role of already established (7–13) or potentially novel and promising molecular biomarkers such as telomere length (14) or microRNAs (15) and additionally to give an overview on the molecular pathology of CRC.

## Conflict of Interest Statement

The author declares that the research was conducted in the absence of any commercial or financial relationships that could be construed as a potential conflict of interest.
